# Small Cell Sarcoma of Nasal and Orbital Cavity, Abscesses of Orbital Cavity and Necrosis of Orbital Plate of Frontal Bone, with Operations

**Published:** 1893-12

**Authors:** A. Bethune Patterson

**Affiliations:** Atlanta, Ga.; Late Assistant to the Royal London Ophthalmic and Golden Square Throat Hospital, London, England


### SMALL CELL SARCOMA OF NASAL AND ORBITAL CAVITY, ABSCESS OF ORBITAL CAVITY AND NECROSIS OF ORBITAL PLATE OF FRONTAL BONE, WITH OPERATIONS.

BY A. BETHUNE PATTERSON, M. D., ATLANTA, GA.

Late Assistant to the Royal London Ophthalmic and Golden Square Throat Hospital, London, England.

Tom Singleton, colored, age 15, was brought to me by his father on April 15, 1889, on account of a protrusion of left eye ball, first noticed about three weeks before, had never experienced any pain in eye or head. The boy was full blooded negro, and general health good. The eye was pressed outwards and forward, fair excursion in all directions, normal pupil—almond shaped, turning easily, full, extending from infra orbital notch below inner canthus. The position of these growths were not changed by the movements of eye. Vision in right eye 6-6, in left eye 6-18, not improved by wearing glasses, optic papilla swollen. The left nasal cavity was completely blocked by a tumor. The nature of the growth was explained to the father and an operation advised, which was agreed to. The growth in nasal cavity was easily removed with forceps and wire snare. Dobell's Solution was ordered as a spray, and the boy sent home to return in ten days to have the tumor removed from orbital cavity. On the 27th of April the boy returned with quite an increase in the exophthalmod, with a marked increase in the size of tumors. The boy was placed under the influence of ether, the brow shaved and bathed in an antiseptic lotion. An incision was made in track of eyebrow and extended into orbital cavity. The flap containing upper lid, including eyebrow was drawn down and out, which gave ample room in which to dissect out the growths, which was soon accomplished by the use of scissor-forceps and handle of scalpel. Care was used in removing each particle of the tumor. The bony margin of opening into nasal cavity was thoroughly curetted, the opening was large enough to admit the end of index finger which came into contact with little finger of left hand passed up nasal cavity. The parts were irrigated with a solution of corrosive sublimate sixteen grains to the pint of warm water. The eye, when released, returned to its normal position. The incision was brought together with silk sutures and dressed antiseptically. The nasal cavity was ordered to be sprayed every few hours with Dobell's Solution. The discharge from nasal cavity at first was quite free, but subsided in a few days. There was a slight elevation of temperature which lasted about twenty-four hours. On the third day the dressing was removed and the stitches taken out, union was perfect, ptosis complete. In a few days he left for home with instructions to continue spray until cessation of discharge. About six weeks after his return home the growth had progressed to a point when operated upon, and as I was away to be absent several months, the boy was taken to a neighboring occulist who operated—enucleating the eye. The patient died suddenly about ten days afterwards. A stained section of the tumor showed it to be a small celled sarcoma.

The latter part of July, 1893 H. K. Reynolds, colored, from Powder Springs, Ga., brought his son, Frank, age 16, full blooded negro, on account of protrusion of right eye, first observed two months ago, gradually growing worse, suffers with pains in head, eye excursus curtailed, no growth could be felt in the rear of eyeball, the latter rested at least half way out of socket, pupil normal, optic papula swollen, vision reduced to 6-24. The right nasal cavity occupied by a large tumor, little space in left on account of hypertrophic tissue, breathes through the mouth. I did not detect the growth in orbital cavity, my diagnosis was sarcoma, and advised immediate removal. The father went away with his son, promising to return the following week. At this writing the boy has not returned. I learn through Dr. R. B. Murray, of Powder Springs, that the boy is still alive.

March 31st 1893, H. P. V., (white,) age 61, Marysville, Ga., farmer and mechanic, came with absess of left orbital cavity, of fourteen years duration, which has incapacitated him from doing almost any kind of work, and unable to care for himself, has spent most of his time in the poorhouse. His history is as follows: Was a Confedrate soldier, served under Lee in the Army of Virginia. During this campaign he suffered constantly with a cold sensation in top of head, which continued until May 1879, when he was taken with a sharp pain in top and left side of head, extending into his eye. Upper lid and surrounding tissues were greatly swollen, soon a discharge was noticed in corner of eye and nose which continued up to May, 1892, when an opening formed in outer third of upper lid, from which pus dropped constantly, the upper lid remained swollen and indurated, through this opening a probe could be easily passed along a short sinus into the orbital cavity, showing a nude and roughened surface. The old man was the picture of wretchedness, in constant pain, emaciated, poorly clad, presenting the symptoms of septicemia, fever, chills, night sweats, etc. On 4th of April ordered a laxative and a bath; 5th 8 A. M. the brow and face scrubbed and shaved, bathed in sublimate solution, conjunctival sac, irrigated with warm boracic acid lotion, patient anæsthetized with chloroform, an incision made through integument at inner angle of supra-orbital arch and parallel to, down and through fibers of orbicularis palpabral into orbital cavity, out of which came a quantity of pus. The probe showed a large excavation and a general roughness of the frontal plate, in fact, from the position of the probe when carried in a vertical position, I am led to believe there was no frontal plate as the probe passed vertically two inches from the supra-orbital notch and horizontally about the same distance. The incision enlarged sufficieutly to admit the little finger, a thorough examination revealed the fact that the frontal plate was intact, though free from periostum, with rough patches here and there, the frontal sinuses were in a healthy condition, the entire frontal plate was affected, there was a crescent like slough of the outer thirds of supra-orbital arch, to the depth of about a fourth of an inch the surface of the excavation was smooth, and therefore did not require scraping. The curette was freely used, making smooth the entire surface of the plate. The cavity was irrigated with a solution of corrosive sublimate and packed with strips of iodoform gauze. The old sinus in the lid was scraped out, a moist antiseptic dressing applied, bandage covered both eyes, soon rallied from the anæsthetic, gave a hypodermic of morphine and put to bed. On the 6th normal temperature, has not slept well, removed dressing and packing, cavity irrigated with sublimate solution, drainage tube inserted and dressing applied; 8th—dressing stained with exudation, removed dressing, washed with sublimate solution and moist dressing applied. As nothing occurred during the treatment worthy of note, I will not fatigue the reader with the daily reports. The dressings were changed once a week up to the 30th of June, when the silver tube which had taken the place of the rubber was removed. The probe showed no filling up of the cavity posteriordly or vertically, the probe passed directly backward a little over two inches, in width I judge the cavity to be about a half inch, showing outer portion of cavity to be filled up, the bony surfaces are completely covered. July 4th—The tube having been left out, there was almost a complete closure, a fullness was complained of which caused me to introduce a hypodermic needle, and draw off about an ounce of a wine colored serous liquid, no evidences of pus. The cavity was thoroughly washed out with peroxide of hydrogen, dressing applied. On 7th again removed a small quantity of serum and washed out with the peroxide of hydrogen until effervescence ceased; 12th—opening closed, needle not used as there was no complaint of fullness, dressing left off; 18th—vision 6-36, there is a slight deviation at a distance, objects are double, the eye appears normal in position and excursion, the lid shows no evidence of its former induration; 25th—discharged, he returned to Marysville, Ga. A recent postal card from him states that he has had no trouble, and his eye is well.

